# Risk factors for intra-abdominal hypertension and abdominal compartment syndrome among adult intensive care unit patients: a systematic review and meta-analysis

**DOI:** 10.1186/cc13075

**Published:** 2013-10-21

**Authors:** Jessalyn K Holodinsky, Derek J Roberts, Chad G Ball, Annika Reintam Blaser, Joel Starkopf, David A Zygun, Henry Thomas Stelfox, Manu L Malbrain, Roman C Jaeschke, Andrew W Kirkpatrick

**Affiliations:** 1 Department of Community Health Sciences (Division of Health Services Research), University of Calgary, Intensive Care Unit Administration, Foothills Medical Center, 3134 Hospital Drive Northwest, T2N 5A1, Calgary, AB, Canada; 2 Departments of Surgery and Community Health Sciences (Division of Epidemiology), University of Calgary, Intensive Care Unit Administration, Foothills Medical Center, 3134 Hospital Drive Northwest, T2N 5A1, Calgary, AB, Canada; 3 Hepatobiliary and Pancreatic Surgery, Trauma and Acute Care Surgery, University of Calgary, Foothills Medical Center, 1403-29 Street NW, T2N 2T9, Calgary, AB, Canada; 4 Clinic of Anaesthesiology and Intensive Care, University of Tartu, Puusepa 8, 51014, Tartu, Estonia; 5 Division of Critical Care Medicine, Zone Clinical Department, Department of Critical Care Medicine, Faculty of Medicine and Dentistry, University of Alberta, General Systems ICU, University of Alberta Hospital, 8440 - 112 Street, T6G 2B7, Edmonton, AB, Canada; 6 Department of Community Health Sciences, University of Calgary, 3280 Hospital Drive NW, T2N 4Z6, Calgary, AB, Canada; 7 Intensive Care Unit and High Care Burn Unit, Ziekenhuis Netwerk Antwerpen, ZNA, Stuivenberg, Lange Beeldekensstraat, 267, 2060, Antwerpen 6, Belgium; 8 Department of Medicine, Department of Clinical Epidemiology and Biostatistics, Critical Care Response Team, St Joseph's Hospital, McMaster University, 50 Charlton Avenue East, L8N 4A6, Hamilton, ON, Canada; 9 The Departments of Surgery and Critical Care Medicine and Regional Trauma Services, Foothills Medical Centre, 1403-29 Street NW, T2N 4Z6, Calgary, AB, Canada

## Abstract

**Introduction:**

Although intra-abdominal hypertension (IAH) and abdominal compartment syndrome (ACS) are associated with substantial morbidity and mortality among critically ill adults, it remains unknown if prevention or treatment of these conditions improves patient outcomes. We sought to identify evidence-based risk factors for IAH and ACS in order to guide identification of the source population for future IAH/ACS treatment trials and to stratify patients into risk groups based on prognosis.

**Methods:**

We searched electronic bibliographic databases (MEDLINE, EMBASE, PubMed, and the Cochrane Database from 1950 until January 21, 2013) and reference lists of included articles for observational studies reporting risk factors for IAH or ACS among adult ICU patients. Identified risk factors were summarized using formal narrative synthesis techniques alongside a random effects meta-analysis.

**Results:**

Among 1,224 citations identified, 14 studies enrolling 2,500 patients were included. The 38 identified risk factors for IAH and 24 for ACS could be clustered into three themes and eight subthemes. Large volume crystalloid resuscitation, the respiratory status of the patient, and shock/hypotension were common risk factors for IAH and ACS that transcended across presenting patient populations. Risk factors with pooled evidence supporting an increased risk for IAH among mixed ICU patients included obesity (four studies; odds ratio (OR) 5.10; 95% confidence interval (CI), 1.92 to 13.58), sepsis (two studies; OR 2.38; 95% CI, 1.34 to 4.23), abdominal surgery (four studies; OR 1.93; 95% CI, 1.30 to 2.85), ileus (two studies; OR 2.05; 95% CI, 1.40 to 2.98), and large volume fluid resuscitation (two studies; OR 2.17; 95% CI, 1.30 to 3.63). Among trauma and surgical patients, large volume crystalloid resuscitation and markers of shock/hypotension and metabolic derangement/organ failure were risk factors for IAH and ACS while increased disease severity scores and elevated creatinine were risk factors for ACS in severe acute pancreatitis patients.

**Conclusions:**

Although several IAH/ACS risk factors transcend across presenting patient diagnoses, some appear specific to the population under study. As our findings were somewhat limited by included study methodology, the risk factors reported in this study should be considered candidate risk factors until confirmed by a large prospective multi-centre observational study.

## Introduction

Intra-abdominal hypertension (IAH) and abdominal compartment syndrome (ACS) are common and associated with substantial morbidity and mortality among critically ill adults [1-4]. These conditions have been linked with acute and chronic renal failure [5-9], multi-organ dysfunction syndrome (MODS) [10], increased lengths of intensive care unit (ICU) and hospital stay [10], and elevated mortality [5,7,10]. Unfortunately, it remains unknown if prevention or treatment (either surgical or medical) of IAH/ACS among these patients improves patient-important outcomes. Identifying critically ill patients at risk for IAH/ACS is therefore important in order to guide identification of the source population for future treatment trials and to stratify patients into risk groups based on prognosis [11].

As clinical examination is likely inadequate for diagnosis of elevated intra-abdominal pressure (IAP) [12,13], trans-bladder pressure monitoring is frequently used to more accurately identify IAH and ACS [14]. The World Society of the Abdominal Compartment Syndrome (WSACS) recommends measuring IAP via the bladder when one or more risk factors for IAH or ACS are present [14]. However, as the risk factors proposed in the latest WSACS guideline were reported to be largely opinion- or pathophysiology-based and occur among nearly all of the patients admitted to the ICU, identifying evidence-based risk factors may better inform IAP screening practices [15].

Although a number of studies of IAH/ACS risk factors have been published, interpretation of their reported estimates of risk is difficult due to significant between-study clinical heterogeneity [1,2,9,10,15-23]. Many of these studies included varying patient populations, ranging from purely medical [1,2,9,15,18,19] to post-operative trauma and other surgical patients [16,17,20,21,23]. Several also used somewhat ambiguous descriptions (for example, blood glucose level [10] and fluid [1,24] or crystalloid resuscitation [1,2,16,17]) or varying thresholds or cutoffs (for example, crystalloid resuscitation >3 L or >7.5 L [17]) to define their proposed candidate risk factors. Finally, some of the studies defined IAH or ACS differently, and adjusted their estimates of risk for potential confounding factors to varying degrees.

In order to assist clinicians in comparing the risk of IAH or ACS development across varying patient populations, risk factor definitions, and study methodologies, we conducted a systematic review of IAH/ACS risk factors among adult ICU patients that utilized a formal narrative synthesis alongside a meta-analysis. As we sought to increase the awareness, dissemination, and use of the findings of this systematic review, we invited international members of the WSACS and WSACS Clinical Trials Working Group to be engaged across all phases of this study.

## Materials and methods

Methods for inclusion of articles and analysis and reporting of their results were specified *a priori* in a protocol developed according to recommendations from the preferred reporting items for systematic reviews and meta-analyses (PRISMA) [25] and the meta-analysis of observational studies in epidemiology (MOOSE) [26] statements. We did not request ethical review of the study as this is not required by the University of Calgary Conjoint Health Research Ethics Board for systematic reviews and meta-analyses.

### Search strategy

We searched Ovid MEDLINE and EMBASE, PubMed, the Cochrane Central Register of Controlled Trials (CENTRAL), and the Cochrane Database of Systematic Reviews from their first available date until January 21, 2013 without restrictions. Two investigators (DJR, AWK) created the initial MEDLINE search strategy. This strategy was then piloted and refined by another investigator (JKH) by adding additional thesaurus/indexing search terms when new and relevant citations were located [27]. Using a combination of Medical Subject Heading (MeSH)/Emtree terms and keywords, we constructed search filters covering the themes IAH/ACS, risk factors, and critical care (see Additional file 1: Table S1 for our final electronic search strategies). In order to identify additional citations, we also used the PubMed 'related articles’ feature, contacted content experts (including members of the WSACS), and manually searched bibliographies of included studies and relevant review articles. Finally, we wrote to the first or corresponding author of nine articles in order to clarify study procedures or obtain additional study data [1,2,9,10,17,20-23].

### Study selection

Two investigators (JKH, DJR) independently screened the titles and abstracts of all identified citations. We used the following inclusion criteria: (1) study participants were adult (≥16 years old) ICU patients; (2) the study reported patient-level characteristics considered candidate risk factors for IAH or ACS; (3) IAH was reported to be diagnosed using trans-bladder pressure measurements [14,28]; (4) odds ratios (ORs) or relative risks (RRs) (either adjusted or unadjusted for potential confounding factors) relating the candidate risk factor with the development of IAH or ACS were provided or could be calculated; and (5) the study design was observational. Although we defined and graded IAH and ACS according to the definitions developed by the WSACS [14], studies using alternate definitions or grading schemes for these conditions were also included. As outlined by the WSACS, primary ACS was considered to be ACS associated with injury or disease in the abdominopelvic region while secondary ACS included that not originating from the abdominopelvic region [14]. Disagreements between investigators regarding study inclusion were resolved by consensus.

### Data extraction

The same two investigators extracted data independently using a pre-designed electronic data extraction spreadsheet piloted on a representative sample of five included studies. We extracted the following data from included studies: (1) design and setting, including ICU type; (2) study participant characteristics, including age, primary patient diagnosis (for example, trauma, intra-abdominal sepsis, or pancreatitis), and severity of illness (for example acute physiology and chronic health evaluation II (APACHE-II) [29], sequential organ failure assessment (SOFA) [30], and injury severity score (ISS) [31]); (3) indications used for IAP measurement; (4) whether the method of trans-bladder IAP measurement followed the recommendations of the WSACS (that is measurement via the bladder at end-expiration in the completely supine position with a maximal instillation volume of 25 mL and the transducer zeroed at the midaxillary line) [14]; (5) whether the patient was mechanically ventilated or breathing unassisted; (6) reported candidate risk factors for IAH or ACS and their exact definitions; and (7) the definition of IAH and ACS.

### Risk of bias assessment

Risk of bias was assessed independently and in duplicate by two investigators (JKH, DJR) using the guidelines proposed by Hayden and colleagues for the evaluation of the quality of prognostic studies [32]. These guidelines assist in evaluating study patient participation and attrition; prognostic factor, outcome, and confounding factor measurement; and the conducted statistical analyses using a four-point ordinal scale (yes/partly/no/unsure) [32]. As the method of statistical data analysis may substantially influence the reported results of an observational study, we also examined whether each of the included observational studies met the more detailed recommendations developed by Moss and colleagues for reporting multivariable logistic regression analyses in the pulmonary and critical care literature [33]. These authors recommend that study investigators report the logistic regression equation developed for the analysis, name the statistical package utilized, identify the selected variables for inclusion in the model, and explain whether attempts were made to assess for inter-variable collinearity, effect measure modification, and model validity [33]. Several other study items of interest were also assessed, including study temporality (prospective versus retrospective), patient enrollment (consecutive versus non-consecutive), the definition of IAH and ACS, and the method of bladder pressure measurement (WSACS versus other) [14]. We also examined whether studies reported the ventilatory status of included patients and whether the patient was calm and/or abdominal muscular contractions were absent during IAP measurements.

### Analysis

Following recommendations provided by Rogers and colleagues and Popay and coworkers on the conduct of narrative synthesis in systematic reviews [34,35], we conducted a staged formal narrative synthesis of candidate risk factors alongside a random-effects meta-analysis.

We began by clustering the identified candidate risk factors for IAH and ACS separately into themes (for example, patient characteristics) and subthemes (for example, the disease severity of the patient as measured by a validated scale) without consideration of presenting patient diagnosis in order to identify those that transcended across patient populations [33]. Within subthemes, we then used vote counting [33] to summarize the direction (that is hazardous or protective) and strength of statistical evidence against the null hypothesis of no risk using a simple ordinal scale. This three-level ordinal scale summarized all identified patient-level characteristics as either a: (1) risk factor (OR point estimate and confidence interval (CI) >1), (2) an exposure that was neither hazardous nor protective (CI included 1), or (3) an exposure that was protective (OR and CI <1) [33].

Vote counting is a relatively novel narrative synthesis tool that can be used to identify patterns across heterogeneous data. It involves pre-determining categories (in this case subthemes) and then assigning 'data points’ (in this case a value of one for each risk factor from a study within a subtheme) to these categories [34,35]. This type of descriptive tool subsequently allows for the creation of simple stacked bar charts, which provide a simple visual representation of how many reported potential risk factors were reported to have a hazardous, null, or protective effect across all of the identified observational studies.

Within subthemes, we then stratified each of the identified candidate risk factors into groupings according to presenting patient diagnosis (for example, trauma, pancreatitis, or mixed ICU patients) [33]. As only a few of the adjusted ORs for the reported risk factors had similar enough definitions (and were estimated from similar populations), only a select number of risk factor estimates could be combined through meta-analysis. However, where appropriate, adjusted risk factor ORs were pooled using random-effects models according to the method proposed by DerSimonian and Laird [36]. As only RRs were available in one study [2], we converted these measures into ORs using the method proposed by Deeks and colleagues [37].

In order to assess for inter-study heterogeneity in our pooled ORs, we calculated Cochran’s Q homogeneity [38] and I^2^ inconsistency statistics [39]. As suggested by Higgins and colleagues, we considered an I^2^ statistic of >25%, >50%, and >75% to represent low, moderate, and high degrees of heterogeneity, respectively [40]. Although we planned to conduct sensitivity or stratified analyses in the presence of inter-study heterogeneity (in order to determine whether our pooled estimates varied across a number of *a priori*-identified covariates), these analyses were only able to be conducted based on patient respiratory status (that is mechanically ventilated versus breathing unassisted) and diagnosis (for example, trauma or severe acute pancreatitis). All analyses were performed using Stata version 12.0 (Stata Corp., College Station, TX, USA).

## Results

### Study selection and characteristics

**Figure 1:**
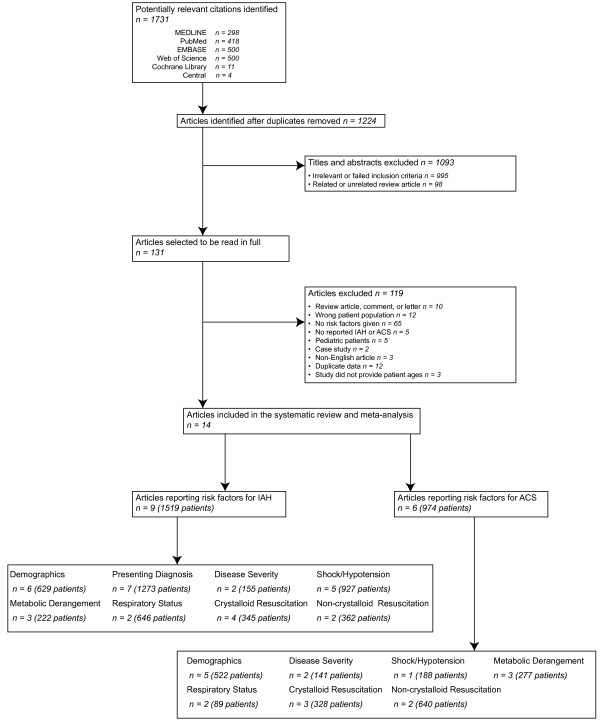
Flow chart of steps in systematic review.

Among 1,224 unique citations, a total of 14 studies enrolling 2,500 critically ill adults met the inclusion criteria (Figure 1) [1,2,9,10,15-24]. Among the 14 included studies, 11 were cohort studies [2,9,10,15-19,22-24], two were case–control studies [20,21], and one was a cross-sectional study [1]. Seven studies included mixed ICU patients [1,2,9,15,18,19,24], one included surgical ICU patients [21], two included severe acute pancreatitis patients [10,22], and four included trauma patients, including those who were in shock [17] or who presented with torso [16], a variety of blunt [23], and severe extremity injuries [20]. Two studies included only mechanically ventilated patients [15,18]. Eight authors [1,9,17,20-23] responded to our requests for supplementary study data, and one [1] provided us with their original dataset and regression modeling strategy such that we could calculate an adjusted OR for a candidate risk factor that was only reported in the manuscript as an adjusted *P* value. The characteristics of the included studies are shown in Table 1.

**Table 1:** Characteristics of studies included in systematic review

**Study, Year**	**Patients (n)**	**Design**	**ICU type**	**Patient population**	**Mechanically ventilated, (%)**	**Reports risk factors for**	**Illness severity**^**a**^
Balogh *et al.*, 2003 [16]	188	Prospective cohort	Trauma	Trauma patients in shock	Not reported	ACS	ISS: 1° ACS^b^ 29 (3)
							2° ACS^b^: 28 (2)
							Normal IAP: 27 (1)
Balogh *et al*., 2011 [17]	81	Prospective cohort	General	Trauma patients in shock	Not reported	IAH	ISS: 29 (1)
Dalfino *et al.,* 2008 [9]	123	Prospective cohort	General	Mixed	Not reported	IAH	IAH, median (IQR): 24 (20–28)
							Normal IAP, median (IQR): 21.5 (18–26)
De Keuleaner *et al.,* 2011 [18]	149	Prospective cohort	General	Mixed mechanically ventilated	100	IAH and ACS	22 (10)
Davis *et al.,* 2013 [22]	45	Retrospective cohort	General	Severe acute pancreatitis	62	ACS	20.3 (6.4)
Ke *et al.,* 2012 [10]	58	Prospective cohort	Surgical	Severe acute pancreatitis	21	IAH	9 (range 8–11)
Kim *et al.,* 2012 [19]	100	Prospective cohort	General	Mixed	81	IAH	19.4 (8.4)
Madigan *et al.,* 2008 [20]	96	Case–control	Trauma	Extremity injury AIS score >3	66	ACS	ISS: ACS 25.6 (9.06)
							Normal IAP: 21.4 (11.02)
Malbrain *et al.,* 2004 [1]	97	Cross-sectional	General	Mixed	Not reported	IAH	SOFA: 6.5 (4)
Malbrain *et al.,* 2005 [24]	265	Prospective cohort	General and specialized	Mixed	Not reported	IAH	17.4 (8.3)
McNelis *et al.,* 2002 [21]	44	Case–control	Surgical	Surgical	100	ACS	ACS: 20.3 (6.5)
							Non-ACS: 11.0 (3.2)
Neal *et al.,* 2012 [23]	452	Prospective cohort	General	Blunt trauma	Not reported	ACS	ISS, median (IQR): 34 (25–43)
Reintam Blaser *et al.,* 2011 [15]	563	Prospective cohort	General	Mixed mechanically ventilated	100	IAH	15.2 (7.4)
Vidal *et al.,* 2008 [2]	83	Prospective cohort	Medical/surgical	Mixed	Not reported	IAH	19 (8)

^a^APACHE II mean (standard deviation) unless otherwise stated; ^b^primary ACS was defined as ACS associated with injury or disease in the abdominopelvic region, secondary ACS was defined as ACS not originating from the abdominopelvic region [14]. ACS, abdominal compartment syndrome; ISS, injury severity score; IAP, intra-abdominal pressure; IAH, intra-abdominal hypertension; IQR, interquartile range; SOFA, *s*equential organ failure assessment; AIS, abbreviated injury scale.

### Risk of bias assessment

While the methodological quality of the included studies was variable, seven satisfied most or all of the criteria proposed by Hayden and colleagues (Table 2) [32]. Although 13 did not report whether abdominal muscular contractions were absent during IAP measurement, one study targeted a Richmond Agitation Sedation Scale score of -5 (unarousable) in all patients undergoing IAP monitoring [18]. Ten studies used the WSACS criteria to define IAH and ACS [1,2,9,10,15,18,19,22],[24,41] while four used alternate criteria to define ACS (including the need for abdominal decompression due to IAP ≥25 mmHg with organ dysfunction, pulmonary/renal/cardiovascular dysfunction, or oliguria/increased peak airway pressure) among some of the included ICU patients (see Additional file 1: Tables S2 and S3) [16,20,21,23]. Further, three studies included a small number of patients diagnosed with IAH/ACS using physical examination alone [20,22,23]. In two of the included 14 studies [15,16], it was unclear if some of the included trauma patients may have been diagnosed with ACS while having an open abdomen as they were reported to have undergone damage control laparotomy.

**Table 2:** **Risk of bias assessment for the included studies**[32]

**Study**	**Participation**	**Attrition**	**Prognostic factor (Risk factors)**	**Outcome measurement (IAH/ACS)**	**Confounding**	**Analysis**
Balogh *et al.,* 2003 [16]	Partly	Unsure	Yes	Partly	Unsure	Yes
Balogh *et al*.*,* 2011 [17]	Partly	Unsure	No	Partly	Unsure	Partly
Reintam Blaser *et al.,* 2011 [15]	Yes	Unsure	Yes	Yes	Unsure	Yes
Dalfino *et al.,* 2008 [9]	Yes	Unsure	No	Yes	Partly	Yes
De Keuleaner *et al.,* 2011 [18]	Yes	Unsure	Yes	Partly	Unsure	Yes
Davis *et al.,* 2013 [22]	Yes	Unsure	Partly	No	Unsure	Yes
Ke *et al.,* 2012 [10]	Yes	Unsure	No	Yes	Partly	Yes
Kim *et al.,* 2012 [19]	Yes	Unsure	Yes	Yes	Unsure	Yes
Madigan *et al.,* 2008 [20]	Yes	Unsure	No	No	Partly	Yes
Malbrain *et al.,* 2004 [1]	Yes	N/A – cross-sectional	Partly	Yes	Unsure	Yes
Malbrain *et al.,* 2005 [24]	Yes	Unsure	Yes	Yes	Partly	Yes
McNelis *et al.,* 2002 [21]	Yes	Unsure	No	Yes	Unsure	Yes
Neal *et al.,* 2012 [23]	Partly	Unsure	Yes	No	Yes	Yes
Vidal *et al.,* 2008 [2]	Yes	Unsure	No	Yes	Unsure	Yes

Where participation was defined as the study sample being representative of the patient population of interest, attrition was defined as loss to follow-up being described and not associated with key participant characteristics (that is, selection bias), prognostic factor indicated that risk factors were adequately defined and measured within the text of the paper (the authors of any studies not satisfying this criteria completely were contacted for definition clarification), outcome measurement indicated that intra-abdominal hypertension/abdominal compartment syndrome (IAH/ACS) is defined and adequately measured (for this category, 'yes’ specifically indicated that the WSACS consensus definitions and guidelines for intra-abdominal pressure (IAP) measurement were used by the studies whereas 'partly’ indicated IAH/ACS that the study used either the WSACS consensus definitions or IAP measurement guidelines (but not both), and 'no’ indicated that either the WSACS consensus definitions or measurement guidelines were both not used, or that some patients had their IAH or ACS diagnosed by physical examination) [42], confounding indicated that potential confounders were appropriately accounted for, and analysis indicated the statistical analysis is appropriate for the study design. In general, for each of the above categories, 'yes’ indicated conditions were satisfied, 'no’ indicated conditions were not satisfied, 'partly’ indicated conditions were partly satisfied, and 'unsure’ indicated it was unclear whether or not conditions were satisfied.

While the quality of reporting of multivariable analyses appeared adequate when assessed using the guidelines proposed by Hayden and colleagues [32] (Table 2), when assessed in more detail using those suggested by Moss *et al*. [33] several methodological limitations became apparent (Additional file 1: Table S4). Eight of the studies failed to provide clearly operational definitions of their reported candidate risk factors (for example, large volume fluid resuscitation) [1,2,9,10,17,20-22]. Moreover, although in all of the included studies except three [10,18,22] the reported ORs were adjusted for potential confounding factors, only two studies specifically reported which covariates were included in the regression model [20,23]. Finally, eight studies [1,9,10,15,16,19,21,22] appeared to select variables for inclusion in the model using stepwise selection procedures (which may have tended to eliminate non-significant factors), none mentioned whether investigators assessed for effect measure modification, and only three reported using a goodness-of-fit test for model validation [2,9,16].

### Risk factors for IAH and ACS

**Figure 2:**
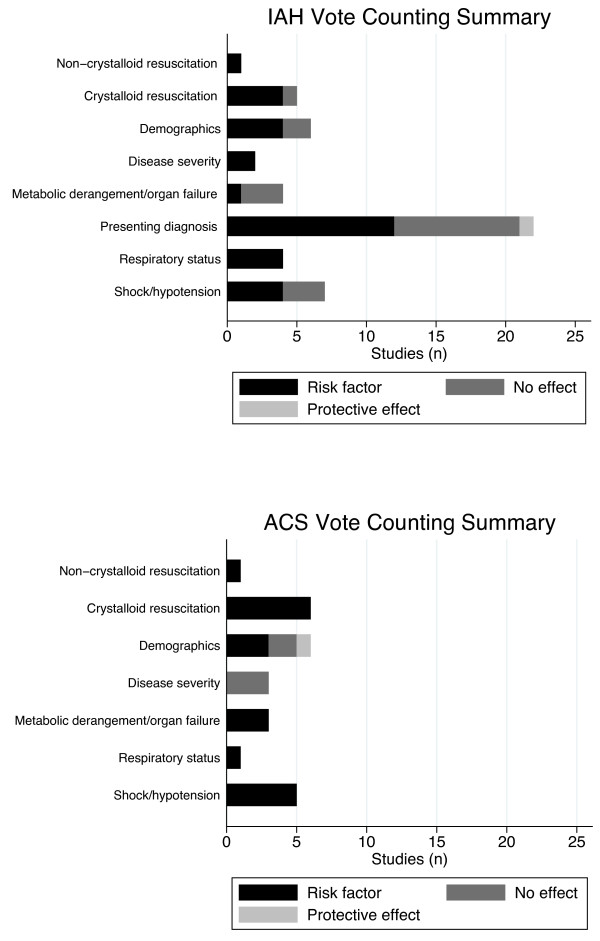
**Vote counting descriptive analysis of candidate risk factors for IAH/ACS.** Vote counting is a descriptive tool that can be used to identify patterns across heterogeneous data. All candidate risk factors from the included studies were first grouped into a subtheme (which is reported along the vertical axis of each of the displayed graphs) and then categorized as to whether they were a: (1) risk factor (odds ratio (OR) point estimate and confidence interval (CI) >1), (2) an exposure that was neither hazardous nor protective (CI included 1), or (3) an exposure that was protective (OR and CI <1) by arbitrarily assigning each of these three categories a representative color (shown in the legend). Subsequently, studies reporting candidate risk factors were assigned a value of one within each of the above-named categories and then summed in order to display the stacked horizontal bar charts shown in each of the vote-counting summary plots. IAH/ACS, intra-abdominal hypertension/abdominal compartment syndrome.

Of the 14 included studies, nine reported candidate risk factors for IAH [1,2,9,10,15,17-19,24] while six reported candidate risk factors for ACS [16,18,20-23]. Using narrative synthesis techniques, we clustered the 62 identified candidate risk factors, including 38 for IAH and 24 for ACS, into three themes, including baseline patient characteristics, systemic physiology, and fluid resuscitation, and eight subthemes (Table 3). The direction of 'risk’ associated with the factors included in each subtheme, and the strength of evidence against the null hypothesis of no risk of IAH/ACS, is summarized in Figure 2. Large-volume crystalloid resuscitation, the respiratory status of the patient, and shock/hypotension were observed to be the most common risk factors for IAH and ACS that transcended across presenting patient populations.

**Table 3:** Narrative synthesis tabulation of candidate risk factor theme and subtheme clusters

**Theme**	**Risk factors included**
**Subthemes**	
**Patient characteristics**	
Baseline demographics	Age
	Gender
	Obesity
	Emergent/surgical status
Presenting diagnosis	Etiology
	Cirrhosis
	Liver dysfunction
	Gastrointestinal bleed
	Ileus
	Sepsis/infections
	White blood cell count
	Abdominal surgery
	Pancreatitis
	Amylase level
	Calcium level
	C-reactive protein level
	Albumin
Disease severity	APACHE-II score
	SOFA score
	Glasgow Coma Scale score
	Revised trauma score
	Injury severity score
	Charlson comorbidity score
	Ranson score
	Glasgow-Imrie score
**Systemic physiology**	
Shock/hypotension	Mean arterial pressure
	Systolic blood pressure
	Shock
	Hypotension
	Vasopressor use
	Capillary leak index
	Central venous pressure
	GAP_CO2_
	Urine output
	Cardiac index
	Hematocrit
	Hemoglobin
Metabolic derangement/organ failure	Base deficit
	Blood glucose
	International normalized ratio
	Hypothermia
	Acidosis
	Serum creatinine
Respiratory status/failure	Respiratory failure
	Acute respiratory distress syndrome
	Mechanical ventilation
	Positive end-expiratory pressure
	Peak airway pressure
	Respiratory rate
**Fluid resuscitation**	
Crystalloid resuscitation	Pre-ICU crystalloid
	Pre-hospital crystalloid
	Emergency department fluid
	24-hour fluid balance
	Fluid balance
	Fluid resuscitation
	Fluid collections
Non-crystalloid resuscitation	Poly-transfusion
	Packed red blood cell units
	Crystalloid: packed red blood cell ratio

APACHE, acute physiology and chronic health evaluation; SOFA, sequential organ failure assessment; GAP_CO2_, gastric mucosal CO_2_ minus end tidal CO_2_.

### Risk factors for IAH

Significant risk factors for IAH (stratified by narrative synthesis subtheme), including those pooled in meta-analysis, are reported in Table 4. In contrast, all IAH risk factors, regardless of their significance level, are reported in (Additional file 1: Table S2).

**Table 4:** Significant risk factors for intra-abdominal hypertension among intensive care unit patients, including pooled estimates where appropriate

**Risk factor**	**Systematic review and/or meta-analysis**					
	**Number**		**Patient population**	**Odds ratio (95% CI)**	**Heterogeneity**	
	**Studies**	**Patients**			**I**^**2**^**, %**	***P *** **value**
**Demographics**						
Obesity [1,15,18,19]	4	909	Mixed ICU patients	5.10 (1.92 to 13.58)	60.2	0.06
Age (per year increase) [9]	1	123	Mixed ICU patients	2.75 (1.01 to 3.09)		
**Presenting diagnosis**						
Sepsis [9,19]	2	223	Mixed ICU patients	2.38 (1.34 to 4.23)	0.0	0.47
Abdominal infection [2,19]	2	183	Mixed ICU patients	2.49 (0.48 to 13.0)	82.7	0.02
Abdominal surgery [2,9,15,24]	4	1034	Mixed ICU patients	1.93 (1.30 to 2.85)	57.1	0.07
Post-laparotomy [17]	1	81	Trauma patients	5.72 (1.50 to 21.43)		
Pancreatitis [15]	1	563	Mechanically ventilated mixed ICU patients	4.73 (1.96 to 11.41)		
Hepatic failure/cirrhosis [15]	1	563	Mechanically ventilated mixed ICU patients	2.07 (2.07 to 28.81)		
GI bleeding [15]	1	563	Mechanically ventilated mixed ICU patients	3.37 (1.43 to 7.94)		
Ileus [2,24]	2	348	Mixed ICU patients	2.05 (1.40 to 2.98)	0.0	0.96
Liver dysfunction [24]^†^	1	265	Mixed ICU patients	2.25 (1.1 to 4.58)		
**Disease severity**						
APACHE II score (per point increase) [10]	1	58	Pancreatitis patients	1.652 (1.131 to 2.414)*		
**Metabolic derangement/organ failure**						
Base deficit [17]	1	81	Trauma patients	1.15 (1.01 to 1.33)		
Acidosis [2]^†^	1	83	Mixed ICU patients	1.93 (1.12 to 3.45)		
**Shock/hypotension**						
Vasopressor use [15]	1	563	Mechanically ventilated mixed ICU patients	2.33 (1.02 to 5.35)		
Shock [9]	1	123	Mixed ICU patients	4.68 (1.93 to 6.44)		
Hypotension [2]^†^	1	83	Mixed ICU patients	2.12 (1.05 to 4.50)		
CVP (per mmHg) [19]	1	100	Mixed ICU patients	1.3 (1.1 to 1.6)		
**Respiratory status/failure**						
PEEP >10cm H_2_0 [15]	1	563	Mechanically ventilated mixed ICU patients	2.41 (1.57 to 3.70)		
Respiratory failure [15]	1	563	Mechanically ventilated mixed ICU patients	1.87 (1.22 to 2.87)		
ARDS [2]	1	83	Mixed ICU patients	3.61 (1.60 to 9.06)		
Mechanical ventilation [2]	1	83	Mixed ICU patients	6.78 (1.94 to 59.03)		
**Crystalloid resuscitation**						
Pre-ICU crystalloid [17]^†,‡^	1	81	Trauma patients	1.40 (1.00 to 1.96)		
Fluid balance [9]^†,‡^	1	563	Mixed ICU patients	5.22 (2.03 to 7.45)		
24hr fluid balance [10]^†,‡^	1	58	Pancreatitis patients	1.004 (1.001 to 1.006)*		
Fluid collections [10]^†^	1	58	Pancreatitis patients	2.015 (1.298 to 3.129)*		
**Non-crystalloid resuscitation**						
Fluid resuscitation (>3.5 L crystalloid or colloid) [1,24]	2	362	Mixed ICU patients	2.17 (1.30 to 3.63)	0.0	0.35

*Unadjusted; ^†^indicative of a risk factor that was not clearly defined; ^‡^although the odds ratio in these studies appeared to increase per liter of fluid, this was unclear in the manuscript. Where abdominal infection was defined as infection of the peritoneal cavity confirmed by radiology or microbiology [19], or pancreatitis, abscess, or other [2]; sepsis was defined according to consensus definitions [9,19]; and respiratory failure was defined as PaO_2_/FiO_2_ <300 mmHg [15]. APACHE II, acute physiology and chronic health evaluation II, CVP, central venous pressure; PEEP, positive end-expiratory pressure; ARDS, acute respiratory distress syndrome.

#### ***Mixed patients***

Among mixed ICU patients, common risk factors for IAH included obesity, sepsis/infection, the presenting diagnosis of the patient, abdominal surgery, acidosis, hypotension, mechanical ventilation/acute respiratory distress syndrome (ARDS), and crystalloid and non-crystalloid resuscitation.

Four studies reported that obesity (defined as body mass index (BMI) >30 kg/m^2^) was a risk factor for IAH in mixed ICU patients [1,15,18,19]. Among these studies, the pooled OR for IAH was 5.10 (95% CI, 1.91 to 13.58; I^2^ = 60.2%; *P* = 0.057). Although this estimate was associated with moderate heterogeneity, one of the four studies included only mechanically ventilated patients [15]. In a sensitivity analysis excluding the results of this study, the pooled OR increased to 8.80 (95% CI, 3.66 to 21.19), and this estimate was homogenous across studies (I^2^ = 0.0%; *P* = 0.945).

Sepsis was also observed to be a risk factor for IAH among mixed populations of ICU patients. Among two studies of mixed ICU patients [9,19], the pooled OR of IAH among those with sepsis was 2.38 (95% CI, 1.34 to 4.23). This estimate was homogeneous across studies (I^2^ = 0.0%; *P* = 0.465).

Four studies reported abdominal surgery as a risk factor for IAH among mixed ICU patients [2,9,15,24]. The pooled odds of IAH among those undergoing abdominal surgery was 1.93 (95% CI, 1.30 to 2.85) times the odds of IAH among patients not undergoing abdominal surgery in these studies. There was a moderate amount of heterogeneity in this estimate (I^2^ = 57.1%; *P* = 0.072), which could not be explained in a sensitivity analysis excluding the results of a single study that included only mechanically ventilated patients.

Two studies [2,24] reported development of ileus to be a risk factor for IAH among mixed ICU patients while two others [1,24] reported large volume fluid resuscitation to be a risk factor (>3.5 L of crystalloid or colloid in the last 24 hours). The pooled OR of IAH associated with ileus development was 2.05 (95% CI, 1.40 to 2.98), which was homogeneous (I^2^ = 0.0%; *P* = 0.960) across studies. The pooled odds of IAH among those who received large volume fluid resuscitation was 2.17 (95% CI, 1.30 to 3.63) times the odds of IAH among those who did not received fluid resuscitation. This estimate was also homogeneous across studies (I^2^ = 0.0%; *P* = 0.350).

#### ***Trauma and severe acute pancreatitis patients***

Among trauma patients, common risk factors for IAH included abdominal surgery, increased plasma base deficit, and pre-ICU crystalloid resuscitation. Among pancreatitis patients, common risk factors included age, gender, disease severity (higher APACHE II and Glasgow-Imrie scores), and large volume crystalloid resuscitation. These risk factors were too clinically heterogeneous to pool through meta-analysis.

### Risk factors for ACS

Significant risk factors for ACS are listed in Table 5. These could often be classified across studies according to location of care (pre-ICU or ICU). All reported risk factors for ACS, regardless of significance level, including those for primary and secondary ACS, are shown in (Additional file 1: Table S3).

**Table 5:** Significant risk factors for abdominal compartment syndrome among intensive care unit patients

**Risk factor**^**†**^	**Systematic review and meta-analysis**			
	**Number**		**Patient population**	**Odds ratio (95% CI)**
	**Studies**	**Patients**		
**Demographics**				
Patient to OR within 75 mins of ED admission [16]	1	188	Trauma patients	102.7 (9.65 to 999.9)
**Disease severity**				
APACHE II score > sample mean of 20.3 [22]	1	45	Severe acute pancreatitis	1.143 (1.012 to 1.292)
Glasgow-Imrie score > sample mean of 9.1 [22]	1	45	Severe acute pancreatitis	1.221 (1.000 to 1.493)
**Metabolic derangement/organ failure**				
Temperature ≤34°C [16]	1	188	Trauma patients	22.9 (1.39 to 378.25)
Hemoglobin ≤80 g/L [16]	1	188	Trauma patients	252.2 (9.89 to 999.9)
Hemoglobin ≤80 g/L [16] (primary ACS)	1	188	Trauma patients	206.1 (7.41 to 999.9)
Base deficit ≥12 [16]	1	188	Trauma patients	3.5 (1.37 to 839.50)
Urine output ≤150 ml in 24 hrs [16]	1	188	Trauma patients	64.1 (5.48 to 749.68)
Serum creatinine > sample mean of 217.7 μmol/L [22]	1	45	Severe acute pancreatitis	1.115 (1.02 to 1.219)*
**Shock/hypotension**				
Systolic blood pressure <86 in ED [16]	1	188	Trauma patients	4.9 (1.78 to 13.99)
GAP_CO2_ ≥16 [16]	1	188	Trauma patients	>999.9 (22.1 to 999.9)
GAP_CO2_ ≥16 [16] (primary ACS)	1	188	Trauma patients	54.3 (2.15 to 999.9)
Urine output ≤150 ml in 24 hrs [16]	1	188	Trauma patients	89.8 (4.49 to 999.9)
Cardiac index <2.6 L/min/m^2^[16]	1	188	Trauma patients	12.5 (1.02 to 153.64)
**Respiratory status/failure**				
Respiratory rate > sample mean of 19.7 breaths/min [22]	1	45	Severe acute pancreatitis	1.004 (1 to 1.008)*
**Crystalloid resuscitation**				
Crystalloid ≥3 L in ED [16]	1	188	Trauma patients	23 (6.38 to 83.10)
Crystalloid ≥3 L in ED [16] (primary ACS)	1	188	Trauma patients	69.8 (10.21 to 477.7)
Crystalloid ≥3 L in ED [16] (secondary ACS)	1	188	Trauma patients	15.8 (1.74 to 143.85)
Crystalloid ≥7.5 L [16]	1	188	Trauma patients	166.2 (4.76 to 999.9)
Crystalloid ≥7.5 L [16] (secondary ACS)	1	188	Trauma patients	38.7 (3.19 to 469.55)
Pre-hospital crystalloid [20]	1	96	Extremity injury patients	1.99 (1.07 to 3.73)
ED crystalloid [20]	1	96	Extremity injury patients	1.85 (1.08 to 3.15)
**Non-crystalloid resuscitation**				
PRBC ≥3 units in ED [16]	1	188	Trauma patients	5.6 (1.03 to 30.83)
Crystalloid:PRBC ratio [23]	1	452	Blunt trauma patients	2.3 (1.4 to 3.8)
Crystalloid (L):PRBCs (units) >1.5:1 [23]	1	452	Blunt trauma patients	3.6 (1.3 to 9.7)

*Unadjusted; ^†^where more risk factors for ACS and primary and/or secondary ACS were reported in a single study, this was indicated in brackets. OR, operating room; ED, emergency department; APACHE II, acute physiology and chronic health evaluation II; ACS, abdominal compartment syndrome; GAP_CO2_, gastric mucosal CO_2_ minus end tidal CO_2_; PRBC, packed red blood cells.

#### ***Pre-ICU ACS risk factors***

Two studies reported pre-ICU (pre-hospital and Emergency Department) risk factors for ACS [16,20]. Pre-hospital crystalloid administration was observed to increase the odds of secondary ACS [20] as did development of a systolic blood pressure <86 mmHg or administration of >3 L of crystalloid in the Emergency Department [16]. Both Emergency Department crystalloid administration and resuscitation with >3 L of crystalloids significantly increased the odds of primary and secondary ACS, while surgical intervention within 75 minutes of Emergency Department admission increased the odds of primary ACS [16].

#### ***ICU ACS risk factors***

Significant risk factors for ACS among those in the ICU included poor disease severity scores, markers of metabolic derangement/organ failure, shock/hypotension, and large volume crystalloid and non-crystalloid resuscitation (Table 5 and Additional file 1: Table S3) [16,22,23]. Among surgical patients, although positive fluid intake/fluid balance were the most common reported candidate risk factors for ACS, these were non-significant when adjusted for other covariates (Additional file 1: Table S3) [21]. In trauma patients, common risk factors similarly included crystalloid and non-crystalloid resuscitation, as well as markers of metabolic derangement/organ failure, and shock/hypotension [16,20,23]. In pancreatitis patients the most common risk factors included high APACHE II and Glasgow-Imrie scores [22]. The varying definitions of these risk factors precluded the production of pooled risk estimates.

## Discussion

Although clinical practice guidelines recommend that IAP be measured via the bladder in all ICU patients with risk factors for IAH or ACS [14], intravesicular pressure measurement is time consuming and may not be required in many ICU patients. When performed at four-hour intervals, IAP measurements may take up to 30 to 42 minutes of nursing time per day [43]. Although controversy exists [44], some have also reported that instillation of saline into the bladder may increase risk of urinary tract infection [41,43]. Thus, defining exactly which patients are at increased risk for IAH and/or ACS could prevent measurement of IAP when not indicated. Further, knowledge of which patient groups are at increased risk for these conditions is important for the design of future studies and to guide clinicians during IAH screening decisions.

This systematic review identified 25 unique significant risk factors for IAH and 16 for ACS. These risk factors could be clustered into three themes, including baseline patient characteristics, systemic physiology, and fluid resuscitation, and eight subthemes. Obesity, certain presenting patient diagnoses (sepsis, intra-abdominal infection, abdominal surgery, pancreatitis, cirrhosis, and gastrointestinal bleeding and ileus), acidemia and hypotension, and large volume crystalloid and non-crystalloid resuscitation were common risk factors for IAH among mixed ICU patients while abdominal surgery, base deficit, and pre-ICU crystalloid resuscitation were common risk factors for IAH among trauma patients. Crystalloid resuscitation was the most common risk factor for ACS in trauma and surgical patients as were indicators of metabolic derangement/organ failure and shock/hypotension. Finally, among patients with severe acute pancreatitis, higher APACHE II/Glasgow-Imrie scores and elevated serum creatinine were risk factors for ACS.

Crystalloid resuscitation prior to ICU admission was found to be a risk factor for both IAH and ACS among trauma patients [16,17,20]. Importantly, although we found that Emergency Department poly-transfusion with packed red blood cells (PRBCs) (≥3 units) was associated with ACS [16], a study by Cotton and colleagues suggests that this risk may be mitigated by use of a massive transfusion protocol that limits crystalloids and provides a larger ratio of plasma and platelets (that is more colloids) [45]. This suggestion is supported by the results of one of the included studies, which reported a graded increase in the odds of ACS as the crystalloid to PRBC ratio increased [23]. Although these findings suggest that there may be an association between the volume or type of administered resuscitation fluids and the incidence of IAH/ACS, the reason for this association remains somewhat unclear as it could be either fluid- or pathology-related. Thus, future studies are needed to determine optimal fluid resuscitation strategies for trauma patients, and IAH/ACS should be considered in these evaluations.

Although this study affords the first comprehensive description of evidence-informed risk factors for IAH/ACS, it has several limitations. First, heterogeneous risk factor definitions and the inclusion of varying patient populations among the included studies precluded the production of pooled OR estimates for many risk factors. Second, as some of the included studies failed to provide clear definitions of their reported risk factors, several likely cannot be readily operationalized in practice. Third, although the quality of reporting of multivariable analyses appeared adequate when assessed using the guidelines proposed by Hayden and colleagues [32], when assessed in more detail using those suggested by Moss *et al*. [33] several methodological limitations became apparent. For example, one study reported an OR upper CI limit that exceeded 1,000 as well as an OR point estimate that was higher than the upper CI limit [16], suggesting that either there were very few events in the logistic regression models used, or the models were highly unstable. Moreover, as stepwise model selection procedures were used by the majority of the included observational studies, some IAH/ACS risk factors may be underreported in this systematic review. Finally, as this study was interested in determining risk factors for IAH and ACS, we excluded studies that examined the effect of exposures on changes in IAP as a continuous measure. Thus, in addition to the risk factors reported in this study, clinicians may also need to consider the influence of other modifiable patient-level variables, including head of bed elevation [46-48] and prone versus supine positioning [49-53].

## Conclusions

In summary, this systematic review and meta-analysis identified 25 unique evidence-informed risk factors for IAH and 16 for ACS. Although several of these risk factors appeared to transcend across patient populations (for example, large-volume crystalloid resuscitation and the presence of shock/hypotension), many were specific to the type of patient population under study. Among mixed ICU patients, certain specific presenting or admission diagnoses, the presence of shock or metabolic derangement, and the volume of crystalloids used in their initial resuscitation appear to be important considerations in determining risk of IAH and ACS. As our findings were partially limited by clinical heterogeneity and the quality of statistical analyses conducted in the included studies, the risk factors reported in this study should be considered candidate evidence-based risk factors until formally evaluated in a prospective multi-centre observational study, which is currently being planned.

## Key messages

• This systematic review and meta-analysis identified 25 unique evidence-informed risk factors for IAH and 16 for ACS

• Although several of these risk factors appeared to transcend across patient populations (for example, large-volume crystalloid resuscitation and the presence of shock/hypotension), many were specific to the type of patient population under study

• Among mixed ICU patients, their specific presenting or ICU admission diagnosis, the presence of shock or metabolic derangement, and the volume of crystalloids used in their initial resuscitation appear to be important considerations in determining risk of IAH and ACS

• Risk factors for IAH with pooled evidence supporting an increased risk among mixed ICU patients included obesity, sepsis, abdominal surgery, ileus development, and fluid resuscitation

• As our findings were partially limited by clinical heterogeneity and the quality of statistical analyses conducted in the included studies, the risk factors reported in this study should be considered candidate evidence-based risk factors until formally evaluated in a prospective multi-centre observational study.

## Abbreviations

ACS: Abdominal compartment syndrome; APACHE II: Acute physiology and chronic health evaluation; BMI: Body mass index; CI: Confidence interval; IAH: Intra-abdominal hypertension; IAP: Intra-abdominal pressure; ICU: Intensive care unit; ISS: Injury severity score; MODS: Multiple organ dysfunction syndrome; MOOSE: Meta-analysis of observational studies in epidemiology; OR: Odds ratio; PRBC: Packed red blood cells; PRISMA: Preferred reporting items for systematic reviews and meta-analyses; RR: Risk ratio; SOFA: Sequential organ failure assessment; WSACS: World Society of the Abdominal Compartment Syndrome.

## Competing interests

The authors declare that they have no competing interests.

## Authors’ contributions

JKH, DJR, CGB, ARB, JS, HTS, DAZ, MLM, RJ, and AWK were involved with study conception and design. JKH, DJR, MLM, and AWK were involved in data acquisition and analysis. JKH, DJR, ARB, JS, HTS, DAZ, MLM, RJ, and AWK interpreted the data and results of the analyses. JKH and DJR drafted the manuscript, which was critically revised for intellectual content by JKH, DJR, CGB, ARB, JS, DAZ, HTS, MLM, RJ, and AWK in serial manuscript revisions. DJR supervised the study. All authors gave approval for the final version of the manuscript to be submitted for publication.

## Authors’ information

JKH is a Masters of Science (Health Services Research) student with a thesis on patient care rounds at the University of Calgary. DJR is a surgery and Clinician Investigator Program resident who is presently conducting a Doctor of Philosophy investigation study in epidemiology with a thesis on trauma damage control surgery at the University of Calgary. CGB and AWK are academic trauma and acute care surgeons while AWK is also an intensivist at the Foothills Medical Center. AWK is also the past President of the Trauma Association of Canada and a member of the Executive Committee of the World Society of the Abdominal Compartment Syndrome. ARB is a member of the World Society of the Abdominal Compartment Syndrome. DAZ is an academic intensivist at the University of Alberta Hospital as well as the Director of the Division of Critical Care Medicine, the Edmonton Zone Clinical Department Head, and a professor in the Department of Critical Care Medicine at the University of Alberta. HTS is an academic intensivist at the Foothills Medical Center and an Associate Professor in the Department of Community Health Sciences at the University of Calgary with expertise in health services research. MLM is the Director of the ICU and High Care Burn Unit at ZNA Stuivenberg Hospital in Belgium. He is also the Past President and Treasurer of the World Society of the Abdominal Compartment Syndrome and the Chairman of Working Group on Abdominal Problems within the European Society of Intensive Care Medicine. RJ is an intensivist and Professor in the Departments of Medicine and Clinical Epidemiology and Biostatistics at McMaster University.

## Supplementary Material

Additional file 1: Table S1 Search strategy; **Table S2.** Reported risk factors for intra-abdominal hypertension (IAH); **Table S3.** Reported risk factors for abdominal compartment syndrome (ACS); **Table S4.** Requirements for reporting of multivariable logistic regression analyses in the pulmonary and critical care literature. Click here for file
