# Supplementary material for: Risk factors for intra-abdominal hypertension and abdominal compartment syndrome among adult intensive care unit patients: a systematic review and meta-analysis
Source: Crit Care. 2013 Oct 21;17(5):R249. doi: 10.1186/cc13075 (PMC4057241; doi:10.1186/cc13075)
Supplement: Additional file 1: Table S1 — Search strategy; Table S2. Reported risk factors for intra-abdominal hypertension (IAH); Table S3. Reported risk factors for abdominal compartment syndrome (ACS); Table S4. Requirements for reporting of multivariable logistic regression analyses in the pulmonary and critical care literature. [file cc13075-S1.docx]

**Risk Factors for Intra-Abdominal Hypertension and Abdominal Compartment Syndrome Among Adult Intensive Care Unit Patients: A Systematic Review and Meta-Analysis**

**Table S1.** Search Strategy.

**Table S2.** Reported Risk Factors for Intra-abdominal Hypertension (IAH).

**Table S3.** Reported Risk Factors for Abdominal Compartment Syndrome (ACS).

**Table S4.** Requirements for Reporting of Multivariable Logistic Regression Analyses in the Pulmonary and Critical Care Literature.

**Table S1.** Search Strategy.

| **Search Theme** | **Search Terms** | | | | |
| --- | --- | --- | --- | --- | --- |
|  | **Ovid MEDLINE, PubMed, and the Cochrane Database** | | **Ovid EMBASE** | | **Web of Science** |
|  | **MeSH Terms** | **Keywords** | **Emtree Terms** | **Keywords** | **Title and Subject Keywords** |
| IAH or ACS | Intra-abdominal Hypertension, Compartment Syndrome | Abdominal compartment syndrome, ((abdominal or intraabdominal or intra-abdominal or bladder or intravesicular or intra-vesicular) adj3 pressure*), ((abdominal or intraabdominal or intra-abdominal) adj3 hypertension) | Intraabdominal Hypertension, Compartment Syndrome, Abdominal Compartment Syndrome, Abdominal Pressure | Abdominal compartment syndrome, ((abdominal or intraabdominal or intra-abominal or bladder or intravesicular or intra-vesicualar) adj3 pressure*), ((abdominal or intraabdominal or intra-abdominal) adj3 pressure*) | Intra-abdominal hypertension, compartment syndrome, abdominal pressure*, intra-abdominal pressure*, intraabdominal pressure, intra-abdominal hypertension, intraabdominal hypertension, bladder pressure, intravesicular pressure, intra-vesicular pressure, abdominal compartment syndrome |
| Risk factors | Risk Factors, Incidence, Risk | Risk marker*, development, risk*, incidence, predict* | Risk Factor, Incidence, Risk, Development | Risk marker*, predict* | Risk factor*, incidence*, risk*, risk marker*, development, predict* |
| Critical care | Critical Care, Critical Illness, Intensive Care, Intensive Care Units | Intensive care, ICU*, critical care, critical illness, critically ill | Intensive Care, Critical Illness, Intensive Care Unit | Critical care, ICU*, critically ill | Critical care, critical illness, intensive care, intensive care unit, ICU*, critically ill |

Abbreviations: IAH, intra-abdominal hypertension; ACS, abdominal compartment syndrome; ICU, intensive care unit

**Table S2.** Reported risk factors for IAH.

| **Study** | **Risk Factor^a^ (definition – if provided in paper or via author through correspondence)** | **Patient Population** | **Definition of IAH: WSACS^b^ or other** | **Point Estimate (95%CI)^c^** |
| --- | --- | --- | --- | --- |
| Balogh *et al.,* 2011 [17] | Base deficit | Shock/trauma patients | WSACS | 1.15(1.01 – 1.33) |
| Balogh *et al.,* 2011 [17] | Post laparotomy state | Shock/trauma patients | WSACS | 5.72(1.50 – 21.43) |
| Balogh *et al.,* 2011 [17] | Pre-ICU crystalloid administration | Shock/trauma patients | WSACS | 1.40(1.00 – 1.96) |
| Reintam Blaser *et al.,* 2011 [15] | Pancreatitis | Mixed mechanically ventilated patients | WSACS | 4.73(1.96 – 11.41) |
| Reintam Blaser *et al.,* 2011 [15] | Hepatic failure/cirrhosis with ascites | Mixed mechanically ventilated patients | WSACS | 2.07(2.07 – 28.81) |
| Reintam Blaser *et al.,* 2011 [15] | Gastrointestinal bleeding | Mixed mechanically ventilated patients | WSACS | 3.37(1.43 – 7.94) |
| Reintam Blaser *et al.,* 2011 [15] | PEEP > 10cmH_2_O | Mixed mechanically ventilated patients | WSACS | 2.41(1.57 – 3.70) |
| Reintam Blaser *et al.,* 2011 [15] | Vasopressor/inotrope administration | Mixed mechanically ventilated patients | WSACS | 2.33(1.02 – 5.35) |
| Reintam Blaser *et al.,* 2011 [15] | Obesity (BMI > 30kg/m^2^) | Mixed mechanically ventilated patients | WSACS | 2.11(1.27 – 3.70) |
| Reintam Blaser *et al.,* 2011 [15] | Laparotomy | Mixed mechanically ventilated patients | WSACS | 2.24(1.47 – 3.42) |
| Reintam Blaser *et al.,* 2011 [15] | Respiratory failure (PaO_2_/FiO_2_ < 300mmHg) | Mixed mechanically ventilated patients | WSACS | 1.87(1.22 – 2.87) |
| Dalfino *et al.,* 2008 [9] | Age (continuous variable) | Mixed ICU patients | WSACS | 2.75(1.01 – 3.09) |
| Dalfino *et al.,* 2008 [9] | Cumulative fluid balance (continuous variable) | Mixed ICU patients | WSACS | 5.22(2.03 – 7.45) |
| Dalfino *et al.,* 2008 [9] | Shock (cardiovascular SOFA subscore > 3) | Mixed ICU patients | WSACS | 4.68(1.93 – 6.44) |
| Dalfino *et al.,* 2008 [9] | Sepsis (defined according to consensus definitions) | Mixed ICU patients | WSACS | 2.11(1.01 – 3.78) |
| Dalfino *et al.,* 2008 [9] | Abdominal surgery | Mixed ICU patients | WSACS | 3.51(1.20 – 6.59) |
| De Keulenaer *et al.,* 2011 [18] | Obesity (BMI > 30 kg/m^2^) | Mixed vechanically ventilated patients | WSACS | 8.0 (2.24 – 43.00)* |
| Ke *et al.,* 2012 [10] | Age (continuous variable) | Severe acute pancreatitis patients | WSACS | 1.006(0.964 – 1.049)* |
| Ke *et al.,* 2012 [10] | Female gender | Severe acute pancreatitis patients | WSACS | 0.440(0.147 – 1.317)* |
| Ke *et al.,* 2012 [10] | Etiology (two physician agreement based on clinical history and lab findings) | Severe acute pancreatitis patients | WSACS | 0.922(0.578 – 1.471)* |
| Ke *et al.,* 2012 [10] | CLI on admission | Severe acute pancreatitis patients | WSACS | 7.140e8(0.012 – 4.380e19)* |
| Ke *et al.,* 2012 [10] | APACHE II (continuous variable) | Severe acute pancreatitis patients | WSACS | 1.652(1.131 – 2.414)* |
| Ke *et al.,* 2012 [10] | 24 hour fluid balance (continuous variable) | Severe acute pancreatitis patients | WSACS | 1.004(1.001 – 1.006)* |
| Ke *et al.,* 2012 [10] | Mean arterial pressure (continuous variable) | Severe acute pancreatitis patients | WSACS | 1.017(0.972 – 1.065)* |
| Ke *et al.,* 2012 [10] | Fluid collections | Severe acute pancreatitis patients | WSACS | 2.015(1.298 – 3.129)* |
| Ke *et al.,* 2012 [10] | Amylase level (continuous variable) | Severe acute pancreatitis patients | WSACS | 1.000(0.999 – 1.000)* |
| Ke *et al.,* 2012 [10] | Hematocrit (continuous variable) | Severe acute pancreatitis patients | WSACS | 9.749(0.009 – 10677.340)* |
| Ke *et al.,* 2012 [10] | White blood cell count (continuous variable) | Severe acute pancreatitis patients | WSACS | 1.045(0.960 – 1.138)* |
| Ke *et al.,* 2012 [10] | Calcium level (continuous variable) | Severe acute pancreatitis patients | WSACS | 0.076(0.010 – 0.579)* |
| Ke *et al.,* 2012 [10] | Blood glucose (continuous variable) | Severe acute pancreatitis patients | WSACS | 0.951(0.828 – 1.093)* |
| Ke *et al.,* 2012 [10] | INR (continuous variable) | Severe acute pancreatitis patients | WSACS | 5.611(0.186 – 168.827)* |
| Ke *et al.,* 2012 [10] | C-reactive protein level (continuous variable) | Severe acute pancreatitis patients | WSACS | 1.005(0.998 – 1.013)* |
| Ke *et al.,* 2012 [10] | Albumin level (continuous variable) | Severe acute pancreatitis patients | WSACS | 0.991(0.867 – 1.131)* |
| Kim *et al.,* 2012 [19] | Obesity (BMI > 30 kg/m^2^) | Mixed ICU patients | WSACS | 8.5(2.7 – 31.9) |
| Kim *et al.,* 2012 [19] | Central venous pressure (factor per mmHg) | Mixed ICU patients | WSACS | 1.3(1.1 – 1.6) |
| Kim *et al.,* 2012 [19] | Abdominal infection (radiologically and/or microbiologically confirmed infection of the peritoneal cavity) | Mixed ICU patients | WSACS | 6.6(1.86 – 26.7) |
| Kim *et al.,* 2012 [19] | Sepsis on admission (defined according to the Society of Critical Care Medicine) | Mixed ICU patients | WSACS | 3.5(1.1 – 11.8) |
| Malbrain *et al.,* 2004 [1] | Obesity (BMI > 30 kg/m^2^) | Mixed ICU patients | WSACS | 12.7 (1.2 – 129.2)** |
| Malbrain *et al.,* 2004 [1] | Total SOFA score | Mixed ICU patients | WSACS | Univariate p value: 0.004  Multivariate p value: 0.310 |
| Malbrain *et al.,* 2004 [1] | Respiratory component of SOFA score | Mixed ICU patients | WSACS | Univariate p value: 0.005  Multivariate p value: 0.212 |
| Malbrain *et al.,* 2004 [1] | Renal component of SOFA score | Mixed ICU patients | WSACS | Univariate p value: 0.002  Multivariate p value: 0.079 |
| Malbrain *et al.,* 2004 [1] | Coagulation component of SOFA score | Mixed ICU patients | WSACS | Univariate p value: 0.020  Multivariate p value: 0.054 |
| Malbrain *et al.,* 2004 [1] | Fluid resuscitation (>3.5L of colloid or crystalloid in the past 24 hours) | Mixed ICU patients | WSACS | 3.3(1.2 – 9.2) |
| Malbrain *et al.,* 2004 [1] | Polytransfusion (>6 units PRBC prior to the study) | Mixed ICU patients | WSACS | 7.3(0.9 – 60.3) |
| Malbrain *et al.,* 2005 [24] | Liver dysfunction | Mixed ICU patients | WSACS | 2.25 (1.1 – 4.58) |
| Malbrain *et al.,* 2005 [24] | Abdominal surgery | Mixed ICU patients | WSACS | 1.96 (1.05 – 3.64) |
| Malbrain *et al.,* 2005 [24] | Fluid resuscitation (>3.5L of crystalloid or colloid in the past 24 hours) | Mixed ICU patients | WSACS | 1.88 (1.04 – 3.42) |
| Malbrain *et al.,* 2005 [24] | Ileus | Mixed ICU patients | WSACS | 2.07 (1.15 – 3.72) |
| Vidal *et al.,* 2008 [2] | Fluid resuscitation | Mixed ICU patients | WSACS | RR: 2.50(0.91 – 6.90) |
| Vidal *et al.,* 2008 [2] | Acidosis | Mixed ICU patients | WSACS | RR: 1.85(1.11 – 3.07) |
| Vidal *et al.,* 2008 [2] | Hypotension | Mixed ICU patients | WSACS | RR: 2.01(1.05 – 3.83) |
| Vidal *et al.,* 2008 [2] | Gastroparesis/Ileus | Mixed ICU patients | WSACS | RR: 1.93(1.23 – 2.99) |
| Vidal *et al.,* 2008 [2] | ARDS | Mixed ICU patients | WSACS | RR: 3.19(1.55 – 6.46) |
| Vidal *et al.,* 2008 [2] | Hypothermia | Mixed ICU patients | WSACS | RR: 1.41(0.75 – 2.63) |
| Vidal *et al.,* 2008 [2] | Mechanical ventilation | Mixed ICU patients | WSACS | RR: 5.26(1.85 – 15.13) |
| Vidal *et al.,* 2008 [2] | Abdominal surgery (previous abdominal surgery) | Mixed ICU patients | WSACS | RR: 1.28(0.93 – 1.75) |
| Vidal *et al.,* 2008 [2] | Abdominal infection (pancreatitis, abscess, or other) | Mixed ICU patients | WSACS | RR: 1.19(0.82 – 1.73) |
| Vidal *et al.,* 2008 [2] | Pneumonia | Mixed ICU patients | WSACS | RR: 1.25(0.85 – 1.85) |
| Vidal *et al.,* 2008 [2] | Bacteremia | Mixed ICU patients | WSACS | RR: 1.13(0.81 – 1.58) |

Abbreviations: IAH, intra-abdominal hypertension; ICU, intensive care unit; PEEP, positive end expiratory pressure; BMI, body mass index; CLI, capillary leak index; APACHE, acute physiology and chronic health evaluation; INR, international normalized ratio; SOFA, sequential organ failure assessment; ARDS, acute respiratory distress syndrome

^a^ all risk factors refer to events occurring in the ICU unless otherwise specified

^b^ odds ratio unless otherwise specified

^c^ WSACS definition of IAH/ACS (grade I, IAP 12-15mmHg; grade II, IAP 16-20mmHg; grade III, IAP 21-25mmHg; grade IV, IAP >25mmHg; and ACS, IAP >20 mmHg associated with new organ dysfunction or failure [14])

*unadjusted

**calculated from original dataset provided by author

**Table S3.** Reported risk factors for ACS.

| **Study** | **Risk Factor^a^ (definition – if provided in paper or via author through correspondence)** | **Risk Factor For** | **Patient Population** | **Definition of ACS: WSACS^b^ or other** | **Point Estimate (95%CI)^c^** |
| --- | --- | --- | --- | --- | --- |
| Balogh *et al.,* 2003 [16] | Crystalloid resuscitation ≥ 3L within 3 hours of ED admission | ACS | Major torso trauma patients | ACS: abdominal decompression performed based on attending trauma surgeon decision in a patient with UBP > 25mmHg with progressive organ dysfunction | 23(6.38 – 83.10) |
| Balogh *et al.,* 2003 [16] | Systolic blood pressure < 86 within 3 hours of ED admission | ACS | Major torso trauma patients | ACS: abdominal decompression was performed based on attending trauma surgeon decision in a patient with UBP > 25mmHg with progressive organ dysfunction | 4.9(1.78 – 13.99) |
| Balogh *et al.,* 2003 [16] | Patient taken to operating room within 75 mins of ED admission | 1°ACS | Major torso trauma patients | ACS: abdominal decompression was performed based on attending trauma surgeon decision in a patient with UBP > 25mmHg with progressive organ dysfunction | 102.7(9.65 – 999.9) |
| Balogh *et al.,* 2003 [16] | Crystalloid resuscitation ≥ 3L within 3 hours of ED admission | 1°ACS | Major torso trauma patients | ACS: abdominal decompression was performed based on attending trauma surgeon decision in a patient with UBP > 25mmHg with progressive organ dysfunction | 69.8(10.21 – 477.7) |
| Balogh *et al.,* 2003 [16] | Crystalloid resuscitation ≥ 3L within 3 hours of ED admission | 2°ACS | Major torso trauma patients | ACS: abdominal decompression was performed based on attending trauma surgeon decision in a patient with UBP > 25mmHg with progressive organ dysfunction | 15.8(1.74 – 143.85) |
| Balogh *et al.,* 2003 [16] | No urgent surgery performed | 2°ACS | Major torso trauma patients | ACS: abdominal decompression was performed based on attending trauma surgeon decision in a patient with UBP > 25mmHg with progressive organ dysfunction | 0.3(0.073 – 0.94) |
| Balogh *et al.,* 2003 [16] | PRBC ≥ 3 units within 3 hours of ED admission | 2°ACS | Major torso trauma patients | ACS: abdominal decompression was performed based on attending trauma surgeon decision in a patient with UBP > 25mmHg with progressive organ dysfunction | 5.6(1.03 – 30.83) |
| Balogh *et al.,* 2003 [16] | GAP_CO2_ ≥ 16 | ACS | Major torso trauma patients | ACS: abdominal decompression was performed based on attending trauma surgeon decision in a patient with UBP > 25mmHg with progressive organ dysfunction | >999.9(22.1 – 999.9) |
| Balogh *et al.,* 2003 [16] | Crystalloid resuscitation ≥ 7.5L within 24 hours of ICU admission | ACS | Major torso trauma patients | ACS: abdominal decompression was performed based on attending trauma surgeon decision in a patient with UBP > 25mmHg with progressive organ dysfunction | 166.2(4.76 – 999.9) |
| Balogh *et al.,* 2003 [16] | Urine output ≤ 150mls within 24 hours of ICU admission | ACS | Major torso trauma patients | ACS: abdominal decompression was performed based on attending trauma surgeon decision in a patient with UBP > 25mmHg with progressive organ dysfunction | 89.8(4.49 – 999.9) |
| Balogh *et al.,* 2003 [16] | Hemoglobin ≤ 8 g/dl | ACS | Major torso trauma patients | ACS: abdominal decompression was performed based on attending trauma surgeon decision in a patient with UBP > 25mmHg with progressive organ dysfunction | 252.5(9.89 – 999.9) |
| Balogh *et al.,* 2003 [16] | Cardiac index < 2.6L/min/m^2^ | ACS | Major torso trauma patients | ACS: abdominal decompression was performed based on attending trauma surgeon decision in a patient with UBP > 25mmHg with progressive organ dysfunction | 12.5(1.02 – 153.64) |
| Balogh *et al.,* 2003 [16] | Temp ≤ 34°C | 1°ACS | Major torso trauma patients | ACS: abdominal decompression was performed based on attending trauma surgeon decision in a patient with UBP > 25mmHg with progressive organ dysfunction | 22.9(1.39 – 378.25) |
| Balogh *et al.,* 2003 [16] | GAP_CO2_ ≥ 16 | 1°ACS | Major torso trauma patients | ACS: abdominal decompression was performed based on attending trauma surgeon decision in a patient with UBP > 25mmHg with progressive organ dysfunction | 54.3(2.15 – 999.9) |
| Balogh *et al.,* 2003 [16] | Hemoglobin ≤ 8g/dl | 1°ACS | Major torso trauma patients | ACS: abdominal decompression was performed based on attending trauma surgeon decision in a patient with UBP > 25mmHg with progressive organ dysfunction | 206.1(7.41 – 999.9) |
| Balogh *et al.,* 2003 [16] | Base deficit ≥ 12 | 1°ACS | Major torso trauma patients | ACS: abdominal decompression was performed based on attending trauma surgeon decision in a patient with UBP > 25mmHg with progressive organ dysfunction | 3.5(1.37 – 839.50) |
| Balogh *et al.,* 2003 [16] | GAP_CO2_ ≥ 16 | 2°ACS | Major torso trauma patients | ACS: abdominal decompression was performed based on attending trauma surgeon decision in a patient with UBP > 25mmHg with progressive organ dysfunction | >999.9(0.001 – 999.9) |
| Balogh *et al.,* 2003 [16] | Crystalloid resuscitation ≥ 7.5L within 24 hours of ICU admission | 2°ACS | Major torso trauma patients | ACS: abdominal decompression was performed based on attending trauma surgeon decision in a patient with UBP > 25mmHg with progressive organ dysfunction | 38.7(3.19 – 469.55) |
| Balogh *et al.,* 2003 [16] | Urine output ≤ 150mls within 24 hours of ICU admission | 2°ACS | Major torso trauma patients | ACS: abdominal decompression was performed based on attending trauma surgeon decision in a patient with UBP > 25mmHg with progressive organ dysfunction | 64.1(5.48 – 749.68) |
| Davis *et al.,* 2013 [22] | Serum creatinine (per unit change above sample mean of 217.7 μmol/L) | ACS | Severe acute pancreatitis patients | WSACS | 1.115(1.02 – 1.219)* |
| Davis *et al.,* 2013 [22] | Respiratory rate (per unit change above sample mean of 19.7/minute) | ACS | Severe acute pancreatitis patients | WSACS | 1.004(1 – 1.008)* |
| Davis *et al.,* 2013 [22] | Age (per unit change above sample mean of 59 years) | ACS | Severe acute pancreatitis patients | WSACS | 0.968(0.922 – 1.017)* |
| Davis *et al.,* 2013 [22] | Obesity (BMI >30 kg/m^2^) | ACS | Severe acute pancreatitis patients | WSACS | 0.56(0.161 – 1.949)* |
| Davis *et al.,* 2013 [22] | Charlson Comorbidity Score (per unit change above sample mean of 2.8) | ACS | Severe acute pancreatitis patients | WSACS | 0.79(0.568 – 1.099)* |
| Davis *et al.,* 2013 [22] | Ranson Score (per unit change above sample mean of 5.4) | ACS | Severe acute pancreatitis patients | WSACS | 1.13(0.798 – 1.601)* |
| Davis *et al.,* 2013 [22] | SOFA Score (per unit change above sample mean of 8.5) | ACS | Severe acute pancreatitis patients | WSACS | 1.079(0.88 – 1.322)* |
| Davis *et al.,* 2013 [22] | APACHE II Score (per unit change above sample mean of 20.3) | ACS | Severe acute pancreatitis patients | WSACS | 1.143 (1.012 – 1.292) |
| Davis *et al.,* 2013 [22] | Glasgow-Imrie Score (per unit change above sample mean of 9.1) | ACS | Severe acute pancreatitis patients | WSACS | 1.211 (1.000 – 1.493) |
| De Keulenaer *et al.,* 2011 [18] | Obesity (BMI > 30 kg/m^2^) | ACS | Mixed mechanically ventilated patients | WSACS | 1.24 (0.20 – 5.58)* |
| Madigan *et al.,* 2008 [20] | Age | 2°ACS | Severe extremity injury patients | ACS: IAP > 25mmHg in the presence of cardiovascular, renal or pulmonary dysfunction.  2°ACS: any patients who received a decompression laparotomy for ACS in the absence of any evidence of primary abdominal injury or had a diagnosis of “secondary ACS” by an attending physician | 1.03(0.99 – 1.07) |
| Madigan *et al.,* 2008 [20] | Male gender | 2°ACS | Severe extremity injury patients | ACS: IAP > 25mmHg in the presence of cardiovascular, renal or pulmonary dysfunction.  2°ACS: any patients who received a decompression laparotomy for ACS in the absence of any evidence of primary abdominal injury or had a diagnosis of “secondary ACS” by an attending physician | 0.94(0.22 – 4.08) |
| Madigan *et al.,* 2008 [20] | GCS | 2°ACS | Severe extremity injury patients | ACS: IAP > 25mmHg in the presence of cardiovascular, renal or pulmonary dysfunction.  2°ACS: any patients who received a decompression laparotomy for ACS in the absence of any evidence of primary abdominal injury or had a diagnosis of “secondary ACS” by an attending physician | 1.10(0.83 – 1.47) |
| Madigan *et al.,* 2008 [20] | Weighted RTS | 2°ACS | Severe extremity injury patients | ACS: IAP > 25mmHg in the presence of cardiovascular, renal or pulmonary dysfunction.  2°ACS: any patients who received a decompression laparotomy for ACS in the absence of any evidence of primary abdominal injury or had a diagnosis of “secondary ACS” by an attending physician | 0.52(0.20 – 1.34) |
| Madigan *et al.,* 2008 [20] | ISS | 2°ACS | Severe extremity injury patients | ACS: IAP > 25mmHg in the presence of cardiovascular, renal or pulmonary dysfunction.  2°ACS: any patients who received a decompression laparotomy for ACS in the absence of any evidence of primary abdominal injury or had a diagnosis of “secondary ACS” by an attending physician | 0.98(0.90 – 1.06) |
| Madigan *et al.,* 2008 [20] | Pre-hospital fluid administration (any crystalloid given before arrival to ED) | 2°ACS | Severe extremity injury patients | ACS: IAP > 25mmHg in the presence of cardiovascular, renal or pulmonary dysfunction.  2°ACS: any patients who received a decompression laparotomy for ACS in the absence of any evidence of primary abdominal injury or had a diagnosis of “secondary ACS” by an attending physician | 1.99(1.07 – 3.73) |
| Madigan *et al.,* 2008 [20] | ED fluid administration (any crystalloid given during the patients time in the ED) | 2°ACS | Severe extremity injury patients | ACS: IAP > 25mmHg in the presence of cardiovascular, renal or pulmonary dysfunction.  2°ACS: any patients who received a decompression laparotomy for ACS in the Madigan *et al.,* 2008[^19^](#_ENREF_19)absence of any evidence of primary abdominal injury or had a diagnosis of “secondary ACS” by an attending physician | 1.85(1.08 – 3.15) |
| McNelis *et al.,* 2002 [21] | 24 hour fluid intake | ACS | Surgical ICU patients | ACS: UBP > 25mmHg with oliguria and increased peak airway pressure | Univariate p value: <0.001  Multivariate p value: >0.05 |
| McNelis *et al.,* 2002 [21] | 24 hour fluid balance | ACS | Surgical ICU patients | ACS: UBP > 25mmHg with oliguria and increased peak airway pressure | Univariate p value: <0.001  Multivariate p value: >0.05 |
| McNelis *et al.,* 2002 [21] | Peak airway pressure | ACS | Surgical ICU patients | ACS: UBP > 25mmHg with oliguria and increased peak airway pressure | Univariate p value: <0.001  Multivariate p value: >0.05 |
| McNelis *et al.,* 2002 [21] | Emergent patient | ACS | Surgical ICU patients | ACS: UBP > 25mmHg with oliguria and increased peak airway pressure | Univariate p value: <0.05  Multivariate p value: >0.02 |
| McNelis *et al.,* 2002 [21] | Base excess | ACS | Surgical ICU patients | ACS: UBP > 25mmHg with oliguria and increased peak airway pressure | Univariate p value: <0.05  Multivariate p value: >0.01 |
| Neal *et al.,* 2012 [23] | Crystalloid to packed red blood cell unit ratio | ACS | Blunt trauma injury patients | ACS: opening of the abdominal cavity for intra-abdominal pressures > 25cm H_2_O with at least one of the following: oliguria (<30mL/h), diminished cardiac output (<2.5L/min/m^2^), elevated static airway pressures (>45cmH_2_O), or P_AO2_/FIO_2_ ratio less than 200. | 2.3(1.4 – 3.8) |
| Neal *et al.,* 2012 [23] | Crystalloid to packed red blood cell unit ratio > 1.5:1 | ACS | Blunt trauma injury patients | ACS: opening of the abdominal cavity for intra-abdominal pressures > 25cm H_2_O with at least one of the following: oliguria (<30mL/h), diminished cardiac output (<2.5L/min/m^2^), elevated static airway pressures (>45cmH_2_O), or P_AO2_/FIO_2_ ratio less than 200. | 3.6(1.3 – 9.7) |

Abbreviations: ACS, abdominal compartment syndrome; ED, emergency department; GAP_CO2_, gastric mucosal CO_2_ minus end tidal CO_2_; ICU, intensive care unit; SOFA, sequential organ failure assessment; APACHE, acute physiology and chronic health evaluation; BMI, body mass index; GCS, Glasgow coma score; RTS, revised trauma score; ISS, injury severity score; IAP, intra-abdominal pressure; UBP, urinary bladder pressure

^a^ all risk factors refer to events occurring in the ICU unless otherwise specified

^b^ odds ratio unless otherwise specified

^c^ WSACS definition of IAH/ACS (grade I, IAP 12-15mmHg; grade II, IAP 16-20mmHg; grade III, IAP 21-25mmHg; grade IV, IAP >25mmHg; and ACS, IAP >20 mmHg associated with new organ dysfunction or failure [14])

*unadjusted

**Table S4.** Requirements for Reporting of Multivariable Logistic Regression Analyses in the Pulmonary and Critical Care Literature [33].

| **Study** | **Summarized the logistic regression equation** | **Statistical packaged named** | **Identified the variables included in the model** | **Specified whether collinearity was assessed** | **Specified whether interaction or effect modification was assessed** | **Stated if model was validated** |
| --- | --- | --- | --- | --- | --- | --- |
| Balogh *et al.,* 2003 [16] | Partly | Yes | Yes | No | No | Yes |
| Balogh *et al.,* 2011 [17] | Partly | No | Partly | No | No | No |
| Reintam Blaser *et al.,* 2011 [[15](#_ENREF_15)] | Partly | Yes | Yes | No | No | No |
| Dalfino *et al.,* 2008 [9] | Partly | No | Partly | No | No | Yes |
| De Keuleaner *et al.,* 2011 [18]* | N/A | N/A | N/A | N/A | N/A | N/A |
| Davis *et al.,* 2013 [22] | Partly | Yes | No | No | No | No |
| Ke *et al.,* 2012 [10] | Partly | Yes | No | No | No | No |
| Kim *et al.,* 2012 [19] | Partly | Yes | Yes | No | No | No |
| Madigan *et al.,* 2008 [20] ^†^ | Partly | Yes | Partly | No | No | No |
| Malbrain *et al.,* 2004 [1] | Partly | Yes | Yes | No | No | No |
| Malbrain *et al.,* 2005 [24] | Partly | No | Yes | No | No | No |
| McNelis *et al.,* 2002 [21] | No | Yes | No | No | No | No |
| Neal *et al.,* 2012 [23] ^†^ | Partly | No | Yes | No | No | No |
| Vidal *et al.,* 2008 [2] | No | Yes | Partly | No | No | Yes |

Where summarized the logistic regression equation indicates the study reported the number of observations, the coefficient of the explanatory variable, an odds ratio and 95% confidence interval for the outcome of interest, and a p-value; statistical package named indicates that the statistical program used was listed somewhere in the paper; identified the variables included in the model indicates variables were properly identified, defined, and coded; specified whether collinearity was assessed indicates that a test for collinearly was used or the concept of collinearity was discussed in the paper; specified whether interaction or effect modification was assessed indicates that the concept of interaction or effect modification was mentioned in the paper; stated if model was validated indicates that a goodness of fit test or another validation technique was used to test the model. Where “yes” indicated conditions were satisfied, “partly” indicated conditions were partly satisfied, “no” indicated conditions were not satisfied, and “unsure” indicated it was unclear whether or not conditions were satisfied.

* N/A (not applicable) as only a univariate analysis was conducted.

^†^ Potential confounding variables considered in these analyses included age, gender, Glasgow Coma Scale, injury severity, shock severity, transfusion/resuscitation requirements, operative interventions, comorbidities, weighted Revised Trauma Score, Injury Severity Score, pre-hospital fluid resuscitation, and Emergency Department fluid resuscitation.
